# Case report: A case of acquired von Willebrand syndrome as onset clinical presentation of systemic lupus erythematosus manifested as epistaxis and pulmonary hemorrhage

**DOI:** 10.3389/fped.2022.1013764

**Published:** 2022-09-20

**Authors:** Songmi Wang, Qun Hu, Yaxian Chen, Xiufen Hu, Ning Tang, Ai Zhang, Aiguo Liu

**Affiliations:** ^1^Department of Pediatrics, Tongji Hospital, Tongji Medical College, Huazhong University of Science and Technology, Wuhan, China; ^2^Department of Clinical Laboratory, Tongji Hospital, Tongji Medical College, Huazhong University of Science and Technology, Wuhan, China

**Keywords:** epistaxis, pulmonary hemorrhage, pediatrics, acquired von Willebrand syndrome, systemic lupus erythematosus

## Abstract

**Background:**

Acquired von Willebrand syndrome (AVWS) is a less common bleeding disorder, primarily manifested as mild to moderate mucocutaneous bleeding and laboratory tests are similar to hereditary von Willebrand disease (VWD). AVWS is secondary to other diseases, and systemic lupus erythematosus (SLE) is a relatively rare cause.

**Case presentation:**

We report a case of AVWS as onset clinical presentation of SLE manifested as epistaxis and pulmonary hemorrhage. A 13-year-old male child presented to the hospital with a six-month history of recurrent epistaxis and a one-month history of anemia. Routine blood tests demonstrated severe normocytic anemia and normal platelet count. Von Willebrand test revealed a significantly lower level. High-resolution chest computed tomography (CT) showed patchy ground glass opacities consistent with hemorrhagic changes. After ruling out the family history, the patient was diagnosed with AVWS. Additional tests confirmed positive antinuclear and anti-Sm antibodies. The underlying SLE was diagnosed and treated with methylprednisolone with disease recovery.

**Conclusion:**

We recommend screening for bleeding disorders in patients with recurrent epistaxis. AVWS should be considered when laboratory findings suggest hereditary von Willebrand disease without a personal or familial history of bleeding. In addition, the underlying disease should be explored.

## Introduction

Acquired von Willebrand syndrome (AVWS) is a rare hemorrhagic disease, similar to hereditary von Willebrand disease (VWD) in laboratory tests and clinical manifestations. AVWS primarily occurs in adults without a personal or family history of bleeding diathesis, characterized by mucocutaneous or gastrointestinal bleeding. Laboratory tests reveal prolonged bleeding time and low levels of plasma factor VIII (FVIII) and von Willebrand factor (VWF) measurements (1). Unlike VWD, AVWS is almost associated with an underlying disease. According to a survey of AVWS by the International Society of Thrombosis and Hemostasis (ISTH) in 2000, among the 186 cases, the associated diseases were lymphatic hyperplasia (48%), myeloproliferative diseases (15%), tumors (5%), immunology (2%), cardiovascular (21%) and other diseases (9%) related (2). Systemic lupus erythematosus (SLE) is a rare cause of AVWS. Herein, we report a case of AVWS as onset clinical presentation of SLE manifested as epistaxis and pulmonary hemorrhage.

## Case presentation

**FIGURE 1:**
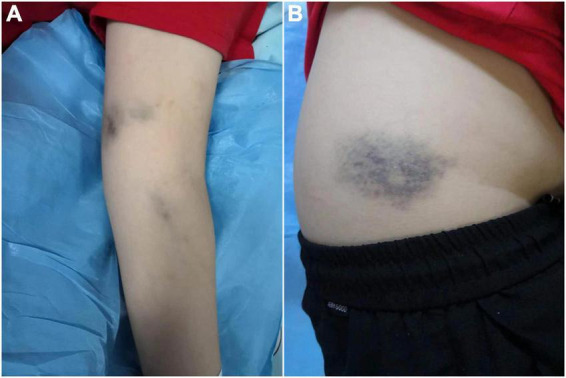
**(A)** Ecchymosis on the upper extremity. **(B)** Subcutaneous hemorrhage on the waist.

A 13-year-old male child without significant past medical history or family history presented to the hematology department with a six-month history of recurrent epistaxis and a one-month history of anemia. Besides, he was prone to be bruised recently. The boy was found to be obese and pallor through physical examination. Old ecchymosis on the extremities and waist, blood scab in the bilateral nasal vestibule but without significant bleeding in the oropharynx could be noticed (Figure 1). Additionally, he had tachycardia (115 beats/min) and hepatosplenomegaly.

On initial laboratory tests, the patient had severe normocytic anemia with hemoglobin (Hb) of 58 g/L (MCV: 87.4fL, MCH: 27.0pg, MCHC: 309g/L, reticulocytes: 2.87%) and normal platelet count. Coagulation function detection showed normal activated partial thromboplastin time (APTT), prothrombin time, and fibrinogen but slightly elevated D-dimer and fibrin degradation products (FDP). As the child had a severe bleeding diathesis, further screening of platelet function tests was performed and turned out to be normal. Subsequent Von Willebrand test revealed a significantly lower level: von Willebrand factor antigen (VWF: Ag) was 19.9%, and von Willebrand factor ristocetin cofactor (VWF: RCo) was 22.7% (Table 1). His parents’ studies were negative for hereditary VWD. Therefore, AVWS was diagnosed.

**TABLE 1:** Laboratory data of the patient during his hospitalization.

	Initial	Post-steroids	Final	Reference values
WBC	5.03	8.95	5.74	3.50–9.5 × 10^9^/L
Hb	58	81	121	130.0–175.0g/L
PLT	246	383	178	125.0–135.0 × 10^9^/L
Reticulocytes	2.87%	/	1.87%	0.5–1.5%
Coombs	++	/	negative	negative
ESR	/	16	72	0–15mm/H
ALT	19	45	24	≤41U/L
AST	34	45	24	≤40U/L
albumin	34	31.2	16.6	32–45g/L
Cr	67	50	42	59–104ummol/L
IgG	29.2	/	4.3	7.0–15.6 g/L
C3	0.18	/	0.37	0.65–1.39 g/L
C4	0.02	/	0.13	0.16–0.38 g/L
ANA	1:3200	/	1:3200	negative
anti-Sm	>8.0	/	>8.0	<1.0
anti-dsDNA	negative	/	1:100	negative
LA	negative	/	negative	negative
PT	14.2	14.5	12.5	12.0–14.5s
APTT	44.9	33.5	40.4	32.0–45.0s
Fibrinogen	3.9	2.56	5.95	2.0–4.0 g/L
VWF:Ag	19.9	56.5	119.2	50–200%
VWF:Rco	22.7	75.4	102.5	50–200%
Hematuria	negative	negative	+	negative
Proteinuria	negative	negative	+++	negative

ALT, alanine aminotransferase; ANA, antinuclear antibodies; anti-dsDNA, anti-double stranded DNA antibodies; anti-Sm, anti-Smith antibody; APTT, activated partial thromboplastin time; AST, aspartate aminotransferase; C3, complement component 3; C4, complement component 4; Cr, creatinine; ESR, erythrocyte sedimentation rate; Hb, hemoglobin; IgG, immunoglobulin G; LA, lupus anticoagulant; PLT, platelet count; PT, prothrombin time; WBC, white blood cell count; VWF:Ag, von Willebrand factor antigen; VWF:RCo, von Willebrand factor ristocetin cofactor. +, positive; ++, moderate positive; +++, strong positive.

**FIGURE 2:**
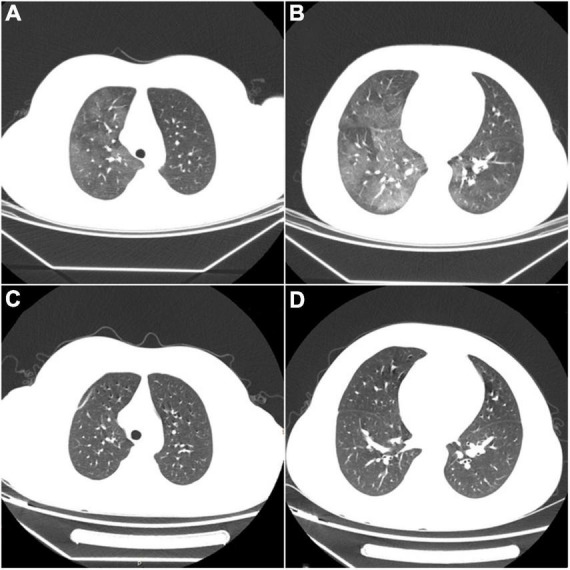
**(A,B)** Diffuse ground-glass opacities consistent with hemorrhagic changes in both lungs. **(C,D)** After treatment, repeated CT shows complete disappearance of the abnormal lesions.

The underlying cause required further investigation. Considering that the patient had normocytic anemia with elevated reticulocytes, the Coombs test was carried out with a positive result. Additional tests confirmed positive antinuclear antibody (1:3200) and anti-Sm antibody. Lupus anticoagulants were negative. The immunologic testing revealed a higher immunoglobulin G level and significantly lower complement levels (C3: 0.18g/L, C4: 0.02g/L). According to Systemic Lupus International Collaborating Clinics (SLICC) classification criteria for SLE (3), the underlying SLE was diagnosed. To further clarify the disease activity index, the following examinations were performed. Although the child had no symptoms such as fever, cough, dyspnea, chest pain, or hemoptysis, lung CT showed diffuse ground-glass nodules consistent with hemorrhagic changes. With the exclusion of infectious lesions, pulmonary manifestations in Systemic Lupus Erythematosus were considered (Figures 2A,B). Urinalysis and color Doppler ultrasound of the urinary system, and magnetic resonance imaging (MRI) of the head, showed no abnormalities. Echocardiography revealed left atrial enlargement and a small amount of pericardial effusion. Mild fatty liver and splenomegaly were found in color ultrasound of the liver and spleen. According to the above, this patient’s systemic lupus erythematosus disease activity index (SLEDAI) (4) was 4 points.

We did not choose desmopressin, VWF-containing concentrates, or intravenous immunoglobulin for hemorrhage control, considering that the child had no acute bleeding or adequate funding. The patient received pulse therapy with methylprednisolone (500 mg/d) for five days, and VWF activity (VWF: Ag 56.5%, VWF: RCo: 75.4%) increased to the normal level. Then the patient had no further active bleeding. In a follow-up to the nine months since he was diagnosed, no further epistaxis or ecchymosis occurred during this period, which was confirmed by his subsequent normal coagulation function and VWF levels. His chest CT also recovered (Figures 2C,D). Unfortunately, the serologic tests suggested higher levels of antinuclear antibodies (1:3200) and strongly positive anti-double-stranded DNA antibodies (1:100). What was worse, his kidneys were affected, shown as nephrotic syndrome included heavy proteinuria (urine protein: + + +, 24-h urinary protein quantification: 5.09g), hypoalbuminemia (16.6g/L), peripheral edema, and hyperlipidemia (total cholesterol 11.97mmol/L, triglycerides 2.30 mmol/L). SLEDAI’s score was as high as 22 points. Renal biopsy showed lupus nephritis (WHO type V).

## Discussion

Acquired von Willebrand syndrome (AVWS) is a less common bleeding disorder, often unrecognized or mistaken for von Willebrand disease. In 1968 Dr. Simone et al. (5) first reported an adolescent with SLE and AVWS. Subsequently, AVWS has regained attention due to its relevance in cardiovascular diseases, including congenital heart defects, aortic stenosis, and using left ventricular assist devices (6). Additionally, AVWS is associated with many other underlying disorders, such as solid tumors, hematological cancers, and autoimmune diseases. More than 20 patients with AVWS and SLE have been reported (7–10).

Most cases with AVWS present mild to moderate mucosal bleeding without a personal or familial history of bleeding. Laboratory tests are similar to hereditary VWD, characterized by prolonged bleeding time and decreased von Willebrand factor levels (e.g., decreased VWF: Ag, decreased VWF: RCo, and/or abnormal high-molecular-weight multimers). FVIII levels may be decreased, and APTT may be prolonged. In this case, the detection of VWF was not performed until six months after the onset of symptoms, despite skin ecchymosis and epistaxis at presentation to the hospital.

Nosebleeds are common in children, with 75% of children experiencing at least 1 episode of epistaxis (11). The nose is well vascularized. The Kiesselbach plexus, located in the anterior nasal cavity, is the main source of epistaxis in children (12). The nasal mucosa provides little anatomical support and protection to the underlying blood vessels, resulting in nasal vascular congestion or any factor that dries or irritates the nasal mucosa can increase the likelihood of epistaxis. Common causes of epistaxis include dry nasal mucosa, sinusitis, trauma, foreign bodies, etc. However, repeated epistaxis should consider the possibility of systemic diseases such as bleeding disorders and tumors (13, 14). Unfortunately, the boy’s parents and the attending physician were likely falsely reassured by his young age and a large amount of exercise.

The pathophysiology of AVWS is not fully understood. Most AVWS patients have normal or even increased VWF synthesis and release, but the removal of VWF from plasma is significantly increased, except for patients with hypothyroidism. Current studies suggest the pathogenic mechanisms underlying AVWS may be related to the following mechanisms: autoantibodies to VWF or FVIII inhibit VWF function and increase the clearance of the FVIII-VWF complex (15); adsorption of VWF by tumor cells, or activated platelets increase the clearance of the FVIII-VWF complex (16); cell-mediated or drug-induced increase proteolysis of VWF multimers (1, 6). Currently, the first mechanism involving circulating antibodies to VWF is considered the primary mechanism of AVWS in SLE patients.

For AVWS, the effective treatment of hemorrhage occurs when the underlying disease and the autoantibodies are controlled (17, 18). Current treatments are mainly for primary diseases and pathogenic mechanisms, including desmopressin, VWF-containing concentrates, intravenous immunoglobulin, recombinant factor VIIa, antifibrinolytics, and plasmapheresis (19–24). For AVWS and SLE, steroids and cyclophosphamide are effective in controlling hemorrhage (25–28). Recent studies have reported that rituximab is used to treat AVWS in adolescent SLE (29–31), and bleeding in both patients has been effectively controlled. In this case, the activity of VWF and FVIII returned to normal levels after methylprednisolone pulse therapy. The primary disease control of the child in a later stage is not ideal, but there is no bleeding diathesis. It suggests that the remission of the primary disease is not always consistent with AVWS (17).

Therefore, it is necessary to perform the relevant examinations for unexplained bleeding. Patients with clinical manifestations and laboratory findings similar to hereditary VWD should be considered AVWS after excluding family history. It is essential to explore the underlying disorders. The key to controlling bleeding is the diagnosis and treatment of the primary disease.

## Data availability statement

The original contributions presented in this study are included in the article/supplementary material, further inquiries can be directed to the corresponding authors.

## Ethics statement

The studies involving human participants were reviewed and approved by the Ethics Committee of Tongji Hospital, Tongji Medical College, Huazhong University of Science and Technology. Written informed consent to participate in this study was provided by the participants’ legal guardian/next of kin. Written informed consent was obtained from the individual(s), and minor(s)’ legal guardian/next of kin, for the publication of any potentially identifiable images or data included in this article.

## Author contributions

SW contributed to the acquisition, analysis of data, and writing the manuscript. NT, YC, AZ, XH, and QH performed the diagnostics and treatment of the patient. AL, QH, and AZ conceived the idea and reviewed the manuscript. All authors have substantively revised the work and approved the final submitted version of the manuscript.

## Abbreviations

APTT: activated partial thromboplastin time
AVWS: acquired von Willebrand syndrome
CT: computed tomography
FDP: fibrin degradation products
FVIII: factor VIII
MRI: magnetic resonance imaging
SLE: systemic lupus erythematosus
SLEDAI: systemic lupus erythematosus disease activity index
SLICC: systemic lupus international collaborating clinics
VWF: von Willebrand factor
VWD: von Willebrand disease
VWF: Ag: von Willebrand factor antigen
VWF: RCo: von Willebrand factor ristocetin cofactor.
